# Atmospheric CO_2_ data from the Australian Grains Free Air CO_2_ Enrichment (AGFACE) facility

**DOI:** 10.1016/j.dib.2022.107937

**Published:** 2022-02-11

**Authors:** Mahabubur R. Mollah, Glenn J. Fitzgerald

**Affiliations:** a Agriculture Victoria, Department of Jobs, Precincts and Regions, 110 Natimuk Road, Horsham, VIC 3400; b Centre for Agricultural Innovation, School of Agriculture and Food, Faculty of Veterinary and Agricultural Sciences, The University of Melbourne, VIC 3010

**Keywords:** SoilFACE, WalpeupFACE, VegeFACE, NFACE, TraitFACE, Australia, Elevated [CO_2_], Climate change

## Abstract

Within the Australian Grains Free Air CO_2_ Enrichment (AGFACE) research program, several facilities were established at different field sites near the towns of Horsham (36.752 S, 142.114 E; 127 m elevation), and Walpeup (35.121 S, 142.005 E; 94 m elevation) in the state of Victoria Australia from 2007 – 2017. These included: TraitFACE, SoilFACE, WalpeupFACE, VegeFACE, and NFACE. These facilities were designed to answer a range of research questions to understand the impacts of elevated CO_2_ (e[CO_2_]) on crop physiology and production. To this end, FACE ‘rings’ (octagons) were built to elevate atmospheric CO_2_ to 550 µmol/mol expected by 2050. These rings were open structures allowing crops to grow freely, without enclosures. Each side of an octagonal ring was individually controlled by a ring-side controller that injected CO_2_ over crops as per the control program. Infrared Gas Analysers (IRGAs) placed at ring centres sampled air continuously from 10 cm above the crop canopy, while CO_2_ was injected at a height 15 cm above the crop canopy. Infrared Gas Analysers (IRGAs) measured atmospheric CO_2_ concentration ([CO_2_]) during the cropping season and provided feedback to the controller to maintain ring-centre [CO_2_] at 550 µmol/mol. The [CO_2_] data were collected from the centre of each FACE ring from 2007 until 2017. The [CO_2_] within a ring was measured each second using calibrated IRGAs. Wind direction and speed were monitored continuously at 2 m above the soil surface at the centre of each ring. These measurements were also collected at the centres of a couple of ambient experimental areas (control – no rings) using the same IRGA and wind sensors. A wireless ethernet local area network (LAN) and a Visual Basic program were used to monitor and transmit data from the individual rings and control areas for data logging. Data at every 4th second and one-minute average (A_MN_CO2) from each ring were logged to daily files, and only A_MN_CO2 data were combined into a seasonal cumulative file. All data recorded during the IRGA warmup period and due to equipment malfunction were removed from cleaned data files. Only A_MN_CO2 data from the rings are uploaded in the Mendeley Data Repository for this article because these data are principally used by scientists and researchers. Data columns in an individual clean file are labelled with abbreviated column names and each file includes: 1) RING, 2) DATE, 3) TIME, 4) A_MN_CO2, 5) REGULAT, 6) WIND_SPD, 7) WIND_DIR and 8) RING_SEC. A limited amount of data (2007 CO_2_ data at ring centres from 8 TraitFACE rings) was published previously [1].

## Specifications Table

**Table:** 

Subject	Engineering
Specific subject area	CO_2_ concentration within the Australian Grains Free Air CO_2_ Enrichment (AGFACE) facility
Type of data	Tables and figures
How data were acquired	We used close path IRGAs (Model SBA-4, PP Systems USA [2]) to measure CO_2_ and Rabbit Semiconductor's microcontrollers with control algorithms written in Dynamic C [3] to control and maintain the release of the appropriate amounts of CO_2_. Wind direction and speed, and [CO_2_] at ring-centres were used by the controller to maintain the target [CO_2_] at 550 µmol/mol at ring-centres. A single controller per ring was used for relatively larger diameter (12 m or 16 m) rings but for smaller diameter rings (4.0 m and 4.5 m), one controller controlled four rings, a modular design for cost-saving. [CO_2_] data were acquired each second by the microprocessor inside the ring controller.
Data format	Raw. Data recorded during the IRGA warmup period and due to equipment malfunction were removed from the cleaned data sets. Data were sorted in Microsoft Excel to delete negative and abnormal values, e.g. 3500 m/s for wind speed.
Parameters for data collection	Parameters of data in each file (Table 1) are described in Table 2 as: 1) RING (identifying the ring number), 2) DATE (date of the year), 3) TIME (time of the day), 4) A_MN_CO2 (one-minute average CO_2_ concentration, 5) REGULAT (electro-pneumatic regulator displaying the extent of valve opening), 6) WIND_SPD (wind speed), 7) WIND_DIR (wind direction) and 8) RING_SEC (main sector of the octagonal ring whose valve is fully open). See Table 3 for an example data file.
Description of data collection	A wireless Ethernet LAN was used to transmit data from the individual AGFACE rings and control (ambient) areas using an Elpro 905U-E modem. Each modem with its own IP address was located within each ring controller box or at a control area to send data, and a single modem of the same model was placed inside the field site office to receive data. Every 4th second and every minute data from each AGFACE ring were sent to an office modem. A Visual Basic program was used for monitoring and logging these data.
Data source location	Institution: Agriculture Victoria (AV), Department of Jobs, Precincts and Regions (DJPR) City/Town/Region: Horsham and Walpeup, Victoria Country: Australia Latitude and longitude for collected samples/data: Horsham (36.752 S, 142.114 E; 127 m elevation), Walpeup (35.121 S, 142.005 E; 94 m elevation) Primary data sources: AGFACE Facilities
Data accessibility	Public repository Repository name: Mendeley Data Data identification number: DOI:10.17632/wcrrtjh37k.1 Direct URL to data: https://data.mendeley.com/datasets/wcrrtjh37k/1 Instructions for accessing these data: Through direct URL Cite this dataset: Mollah, Mahabubur; Fitzgerald, Glenn (2021), “AGFACE [CO_2_] Dataset”, Mendeley Data, V1, DOI: 10.17632/wcrrtjh37k.1
Related research article	Mollah MR, Norton RM, Huzzey J (2009) Australian Grains Free Air Carbon dioxide Enrichment (AGFACE) facility: design and performance. Crop & Pasture Science 60, 697-707. doi:10.1071/CP08354.

## Value of the Data

• These data [4] are useful for the design and performance of Free Air CO_2_ Enrichment (FACE) systems which inject pure CO_2_ from individually controlled horizontal tubes (sectors of an octagon) surrounding FACE rings (octagons). It will provide data to assess the variation and stability of [CO_2_] at ring centres. Models for wind speed and direction vs [CO_2_] can be developed from these data as co-variates to analyse biophysical data.
• Engineers and scientists who design and develop FACE systems will benefit from these long-term data that were collected over 11 years.
• Year-to-year [CO_2_] data at ring-centres can be analysed to evaluate the performance of pure CO_2_ injected FACE rings for various ring sizes and wind speeds. This will help engineers and scientists to optimise the FACE design.

## 1 Data Description

All data are in text files and details are specified in Table 1. Column titles are described in Table 2. Example data are presented in Table 3. File naming and organisation was guided by the International Consortium for Agricultural Systems Applications (ICASA) Version 2.0 [5]. Only one-minute average e[CO_2_] data from the centre of each FACE ring are uploaded in the Mendeley Data Repository for this article because these data are principally used by scientists and researchers. These data are not corrected for daylight savings time. The target [CO_2_] at the ring-centre was set at 550 µmol/mol, which is the concentration predicted for 2050 [6]. Values fluctuated around this target, maintaining the average close to the target. Data were logged from the date when CO_2_ injection began until the date when CO_2_ injection stopped. In 2015, the season started approximately in mid-June and finished near the end of November. However, the data after 10 August 2015 are lost.

**Table 1:** Details of one-minute average e[CO_2_] (measured at ring-centre) data files uploaded on to Mendeley Data Repository. The AGFACE facility name, location, ring experimental diameter and year of the experiment are listed.

File Name for Mendeley Data Repository	AGFACE Facility	Location	Ring/Exp. Area Diameter (m)	Year
ECO2_MIN_AVRHOR2007001.txt	TraitFACE	Horsham	12	2007
ECO2_MIN_AVRHOR2008001.txt	TraitFACE	Horsham	12	2008
ECO2_MIN_AVRWAL2008001.txt	WalpeupFACE	Walpeup	4	2008
ECO2_MIN_AVRHOR2009001.txt	TraitFACE	Horsham	16	2009
ECO2_MIN_AVRHOR2009002.txt	SoilFACE	Horsham	4.5	2009
ECO2_MIN_AVRWAL2009001.txt	WalpeupFACE	Walpeup	4	2009
ECO2_MIN_AVRHOR2010001.txt	TraitFACE	Horsham	16	2010
ECO2_MIN_AVRHOR2010002.txt	SoilFACE	Horsham	4.5	2010
ECO2_MIN_AVRHOR2010003.txt	VegeFACE	Horsham	4	2010
ECO2_MIN_AVRHOR2011001.txt	TraitFACE	Horsham	16	2011
ECO2_MIN_AVRHOR2011002.txt	SoilFACE	Horsham	4.5	2011
ECO2_MIN_AVRHOR2012001.txt	TraitFACE	Horsham	16	2012
ECO2_MIN_AVRHOR2012002.txt	SoilFACE	Horsham	4.5	2012
ECO2_MIN_AVRHOR2013001.txt	TraitFACE	Horsham	16	2013
ECO2_MIN_AVRHOR2013002.txt	SoilFACE	Horsham	4.5	2013
ECO2_MIN_AVRHOR2014001.txt	TraitFACE	Horsham	12	2014
ECO2_MIN_AVRHOR2014002.txt	SoilFACE	Horsham	4.5	2014
ECO2_MIN_AVRHOR2015001.txt	NFACE	Horsham	12	2015
ECO2_MIN_AVRHOR2015002.txt	TraitFACE	Horsham	12	2015
ECO2_MIN_AVRHOR2015003.txt	SoilFACE	Horsham	4.5	2015
ECO2_MIN_AVRHOR2016001.txt	NFACE	Horsham	12	2016
ECO2_MIN_AVRHOR2016002.txt	SoilFACE	Horsham	4.5	2016
ECO2_MIN_AVRHOR2017001.txt	NFACE	Horsham	12	2017

**Table 2:** Column titles for each file uploaded in the Mendeley Data repository with data description and unit of measure.

Column ID	Abbreviated Column Name	Description	Unit or type
1	RING	Ring number	Text
2	DATE	Data collection date	DD/MM/YYYY
3	TIME	Time of data collection	24-hour time
4	A_MN_CO2	One-minute average CO_2_ concentration	µmol/mol
5	REGULAT	Electro-pneumatic regulator	Number
6	WIND_SPD	Wind speed measured	m/s
7	WIND_DIR	Wind direction	Degree
8	RING_SEC	Main sector of the octagonal ring whose valve is fully open	Number (see text)

**Table 3:** Example data table.

RING	DATE	TIME	A_MN_CO2 (µmol/mol)	REGULAT (0-4)	WIND_SPD (m/s)	WIND_DIR (°)	RING_SEC
9	20/07/2008	7:45:36	613	0.17	1.8	79	2
9	20/07/2008	7:46:36	607	0.13	1.8	78	2
9	20/07/2008	7:47:36	563	0.15	1.9	76	2
9	6/09/2008	11:28:51	612	1.03	1.3	227	5
9	6/09/2008	11:29:51	569	0.97	1	193	3
9	6/09/2008	11:30:51	546	1.06	1.3	207	5
9	6/11/2008	17:27:09	571	1.88	2.6	256	6
9	6/11/2008	17:28:09	566	1.92	2.7	251	6
9	6/11/2008	17:29:09	555	1.89	2.9	248	5

At the beginning of each season, all IRGAs were calibrated to 550 µmol/mol [CO_2_] using laboratory standard calibration gas.

Description of Tables and Figures are listed below in the order they appear in the article.

**Fig. 1:**
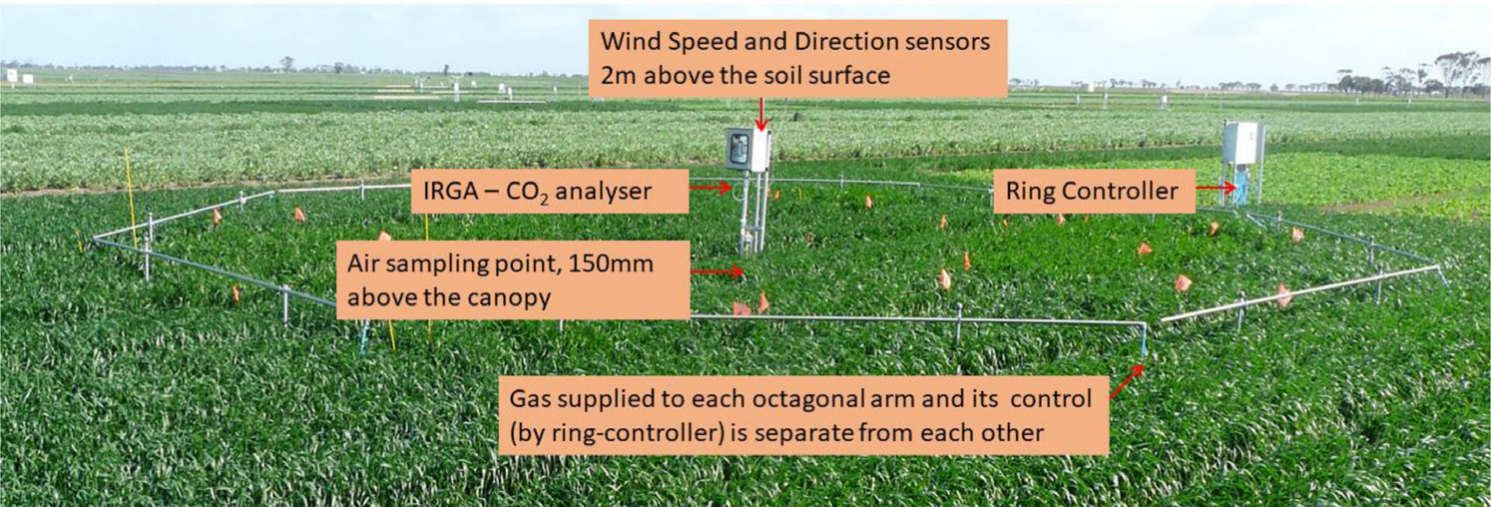
A TraitFACE octagonal ‘ring’ and its components (photo by Rob Norton).
• Fig. 2 shows an example of a typical plot layout used in 2009 for experiments at Horsham. There were 24 plots (∼1.5 × 4 m) per ring split for water (irrigated or rainfed) with a range of wheat varieties and treatments. The IRGA for measuring [CO_2_] was located in the ring centre, in the walkway between plots.

• Table 1 shows the description of one-minute average e[CO_2_] (measured at ring-centre) raw data files which are published on to Mendeley Data Repository. It includes the year, AGFACE facility, location, ring diameter and year of the experiment.
• Table 2 displays the eight abbreviated column titles (parameters) that are used in each data file (refer to Table 1) which are published in the Mendeley Data Repository. It also provides a description of each abbreviation and the nature and unit of the parameters.
• Table 3 provides an example of a data table in the Mendeley Data Repository with column headings showing typical data in the months of July, September and November 2008 over the growing season.
• Fig. 1 shows wheat grown inside a ring. This figure also shows the locations of the main components of the ring which are: Ring controller (outside of the ring); an infrared gas analyser (IRGA) to analyse [CO_2_] at ring-centre; wind speed and direction sensors placed 2 m above the soil surface at ring-centre; air sampling location at the ring-centre and gas being supplied individually to each sector of the octagonal ring.

**Fig. 2:**
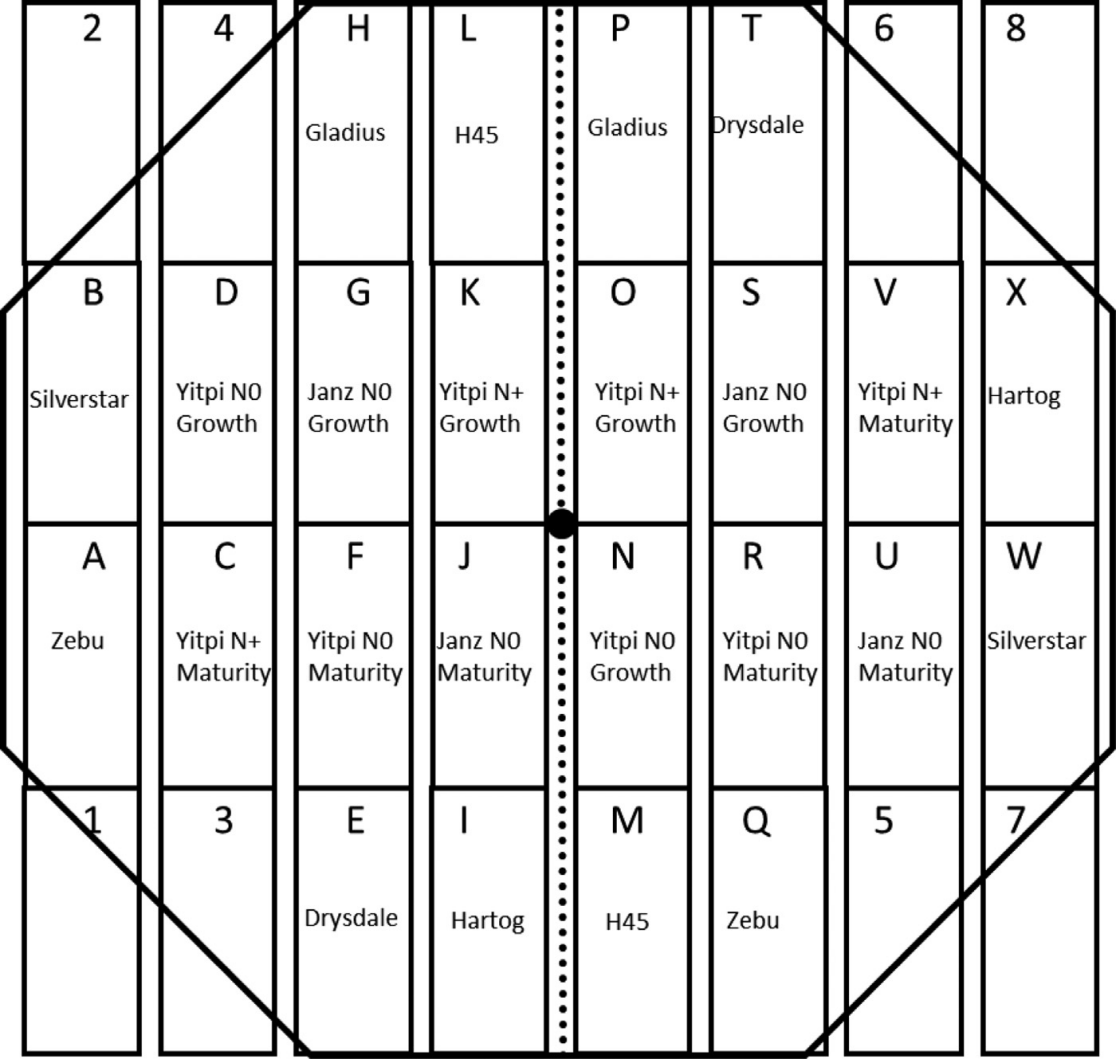
Example plot layout for 2009 experiments in Horsham. A ring split for irrigation level (dotted line). Plots (rectangles) were sown to different crop cultivars. The black circle in the centre shows the location of IRGA. (Modified from Fitzgerald *et al.*[8]).
• Fig. 3 displays a typical SoilFACE array where different crops were grown inside intact soil cores maintaining the physicochemical integrity of the soil profile. There were three different types of soils in each ring. Fig. 3 shows the components for injecting CO_2_ gas to elevate gas concentration above ambient inside the ring (like TraitFACE, Fig. 1).

**Fig. 3:**
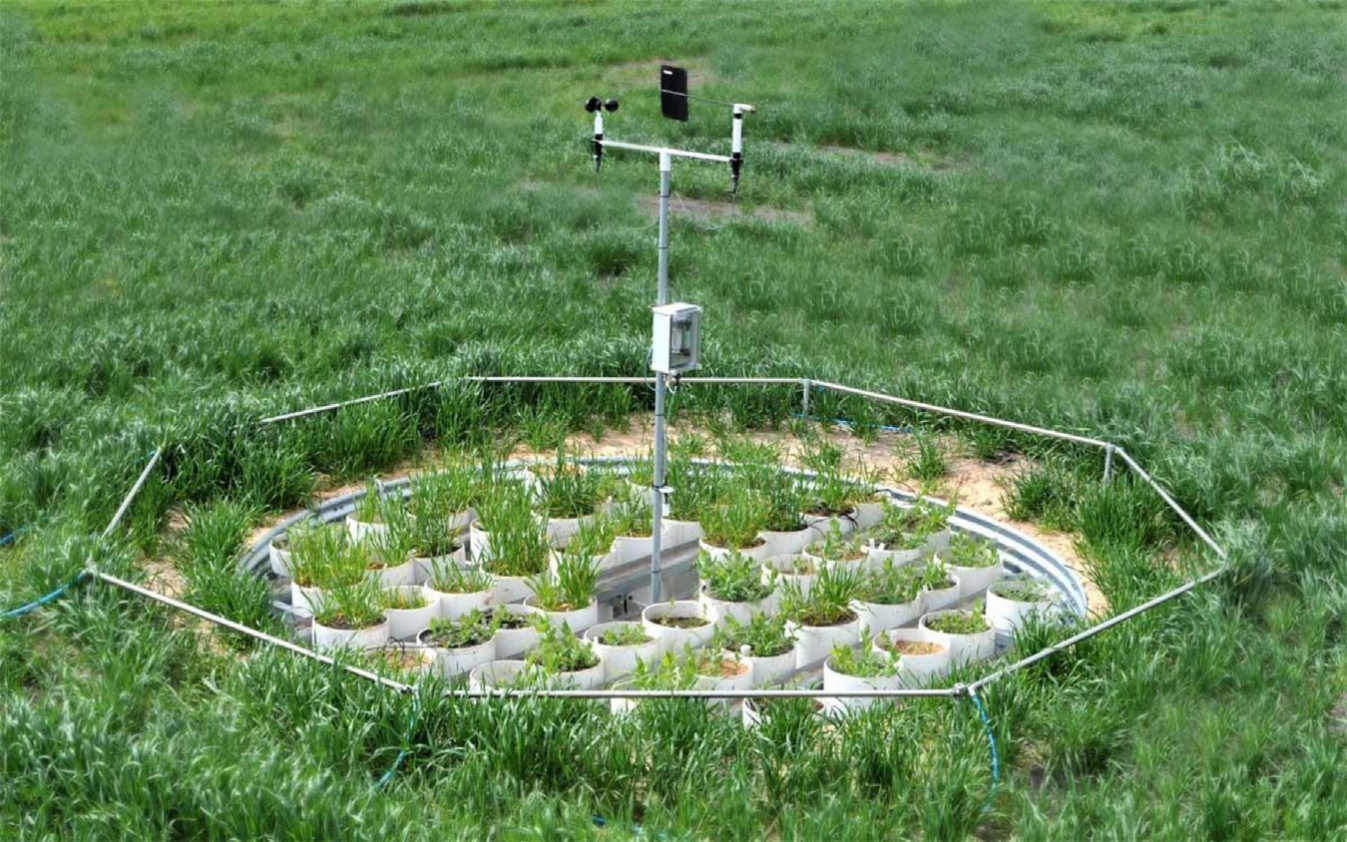
SoilFACE array at Horsham (2009 – 2016) (Photo by Mahabubur Mollah).
• Fig. 4 displays a VegeFACE ring with instrumentation to measure the spatial distribution of CO_2_ inside the ring.

**Fig. 4:**
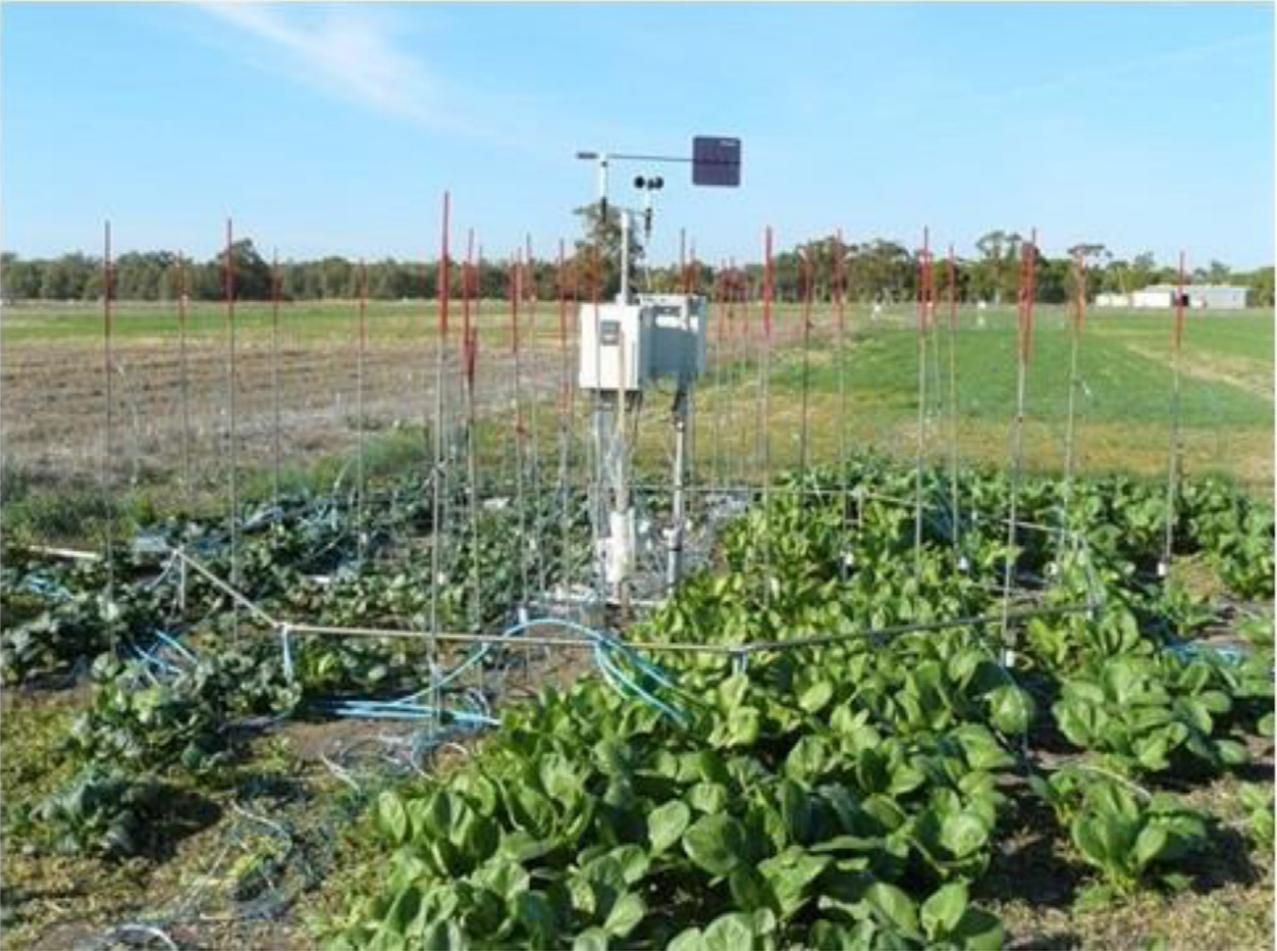
VegeFACE at Horsham (2010) (Photo by Mahabubur Mollah).

## 2 Experimental Design, Materials and Methods

AGFACE facilities were outdoor laboratories (see Fig. 1 for an example) where the impacts of expected e[CO_2_] in the year 2050 (550 µmol/mol) on crops were examined [1,^7^,^8^,^9^,^10^,11]. The AGFACE facilities were built to understand the impacts of different combinations of irrigation, nitrogen fertilizer and time of sowing (TOS) treatments at two locations and across different soil types under e[CO_2_] on a range of crops [9]. These data have supported analysis to better understand agronomic and physiological impacts to agricultural production, forecasting of impacts to future production, and biophysical modelling. Each facility was given a unique name based on the experimental objectives, to evaluate grain crops under e[CO_2_]: SoilFACE to evaluate crops and soil interactions under e[CO_2_]; VegeFACE to evaluate vegetable crop under e[CO_2_]; TraitFACE to evaluate the response of various wheat traits under e[CO_2_]; NFACE to evaluate nitrogen management under e[CO_2_]; WalpeupFACE to evaluate the impact of e[CO_2_] on wheat grown in a relatively drier and hotter site (established in Walpeup, a town in the Mallee region of north-west Victoria Australia which is drier and hotter than Horsham, where the other facilities were located [3], [8], [9]).

The rings in each facility were separated by at least 5.5 ring diameters to avoid wind-blown CO_2_ contamination to the non-CO_2_ controls. This spacing protocol between rings was an unpublished rule-of-thumb adopted by the FACE community including in Australia [1]. All AGFACE facilities had provision for the storage and reticulation of bulk liquid CO_2_ to supply the rings with CO_2_ gas as well as electricity and water to service each part of the experiment. The injection of CO_2_ into the rings began at crop emergence and ceased at crop maturity, covering the photosynthetically active crop stages and only occurring in the day.

In all Free Air CO_2_ Enrichment (FACE) systems, pure CO_2_ was injected into the atmosphere on the upwind side of each ring through 0.30 mm holes (20 holes/m) drilled by laser on 23-mm i.d. stainless-steel tubes at supply line pressures up to 500 kPa. The CO_2_ was released against the wind, which then quickly mixed with air and was transported across the ring by the prevailing wind. The rate of gas flow was pressure-dependent, which varied depending on wind speed, wind direction and [CO_2_] level at the ring centre. The ‘ring’ in this design was an octagon (Fig. 1) and one of the key elements of the design was that gas flow to each side (sector) of the ring was controlled by a separate proportional valve, see section 4.0 in [3].

The IRGA for each ring measured trace gas by determining the absorption of an emitted infrared light source through air samples. Air from ring-centres and control areas was continuously drawn through the IRGA by an air pump. The air sampling inlet (Fig. 1) was placed 5 cm below the CO_2_ fumigation tubes (which were placed 15 cm above the crop canopy) to make sure the sampled air was the true representation of [CO_2_] inside the ring. Air to the IRGA was dried by drawing it through a 50 mm diameter non-sterile syringe filter with a 1 µm filter pore size and holdup volume capacity < 0.1 mL, (see Appendix A in [3]) before passing through a soda-lime absorber column.

A single controller per ring was used for relatively larger diameter (12 m or 16 m) rings such as TraiFACE and NFACE but for smaller diameter (4.0 m and 4.5 m) rings like SoilFACE and WalpeupFACE, one controller controlled four rings, a modular design for cost-saving.

The electro-pneumatic regulator of the ring sector facing the wind perpendicularly, opened fully (100%) and the adjacent ones at 40%, see section 4.10 in [3]. The controller was programmed to cease CO_2_ injection when the wind speed averaged over 45 s at the set maximum speed target. All sectors injected CO_2_ equally at a lower rate when wind speed was <0.3 m/s. Carbon dioxide was only released from the time of sunrise to sunset because crops are not photosynthetically active at night and for cost-saving. The overview of the AGFACE system, controllers and data acquisition methodologies are described in detail elsewhere [1], [3].

### 2.1 TraitFACE

The AGFACE facility at Horsham was arranged as a factorial split-split plot design with four blocks (replications). In each replicate, there were two experimental main plots (‘rings’); one ring had e[CO_2_] at 550 µmol/mol and the other was an ambient experimental plot (a[CO_2_]) at prevailing atmospheric [CO_2_] (384-407 µmol/mol over the 11 years). The areas immediately around plots were sown to wheat to act as a buffer. Each year, the plots were relocated to adjacent areas, so wheat was not grown consecutively on the same plot of land to minimise the possibility of soil-borne root disease [8]; except from 2010-2013 in Horsham, when the experiment was set up as a wheat-field pea rotation and the rings were left in the same locations. In 2007, plots were split in half and each half randomly assigned for two times of sowing (TOS) while from 2008 – 2013 each plot was split in half along the north-south axis by inserting a plastic barrier to 0.8 m depth to separate the Rainfed and Irrigated treatments. Irrigation treatments were randomly allocated to one half of each plot. Cultivars were allocated to areas within each half of the plots. In 2007 and 2008, the plots were 12 m in diameter, and in 2009, the plots were expanded to 16 m and additional cultivars added, again from 2014 to 2017 the plots were reduced to 12 m including for TraitFACE and NFACE. See Fig. 2 for an example plot layout. From 2014 – 2015, the response of different lines of wheat carrying particular traits such as: tillering, transpiration use efficiency, carbohydrate storage, nitrogen use efficiency, rooting, and grain protein were tested using e[CO_2_].

### 2.2 WalpeupFACE

At Walpeup, the experiment was arranged as a randomized complete block with four replications and eight e[CO_2_] plots (rings) and eight a[CO_2_] plots. Each ring was 4.0 m diameter and had separate plots for growth and maturity sampling (see Fig. 3 in [8]). Plots were separated by 25 m within a field of wheat (cv Yitpi). Treatments were two TOS, and two [CO_2_] levels, with the same two levels of [CO_2_] (ambient and 550 µmol/mol), by two TOS as at Horsham from 2007-2009. The crop sown was wheat (cv. Yitpi). Supplementary irrigation was applied as needed to the whole experiment to provide sufficient water to the crop to achieve a harvestable yield, but there were no additional water or N treatments [8], [9]. In 2009, the experiment was shifted to an adjacent area, which was sown to canola (*Brassica napus*) between seasons to avoid any soil disease carryover. For more information on WalpeupFACE, consult references, 3, 8 and 9.

### 2.3 SoilFACE

In SoilFACE, the focus was on how different soil types interacting with e[CO_2_] affected crop growth. In SoilFACE (Fig. 3) intact soil cores were collected by inserting large (30 cm diameter × 100 cm deep) PVC sleeves into the soil. This method assured that each sample was an intact, undisturbed soil core maintaining the physicochemical integrity of the soil profile. Cores were collected from the Victorian Mallee (Calcarosol, pH 5.9), Wimmera (Vertosol, pH 7.7) and High Rainfall Zone (Chromosol, pH 4.50) [10]. The experiments at the SoilFACE commenced in 2009 and finished in 2016. The cores were placed in eight bunkers sunk into the ground with the top of the cores flush with the ground level (Fig. 3). Four bunkers were open to a[CO_2_] over the experimental period whilst four others received e[CO_2_] maintained at 550 µmol/mol [CO_2_] at ring-centres. Rings were 4.5-m in diameter. Experiments assessed the impacts of e[CO_2_] on soil microbiology, nitrogen mineralisation rates, and the amount of nitrogen and carbon distributed belowground in the soil while growing wheat, canola and legume crops.

### 2.4 VegeFACE

In 2010, the TraitFACE setup was adapted during the off-season (January to April), for the smaller diameter VegeFACE rings, resulting in one controller per 4.0 m diameter rings. Eight rings of 4-m diameter were positioned centrally within a 5 × 5 m irrigated zone (Fig. 4). Leafy Asian Brassica cultivars ‘Karate’ and ‘Chop Suey’ were sown in each ring (one variety to one half of the ring). Eight plots under e[CO_2_] treatment (4-m diameter rings) were established along with eight a[CO_2_] control plots. The experiment measured the interacting effects of e[CO_2_], nitrogen (low/high) and two *Brassica rapa* cultivars on shoot growth under south-eastern Australian conditions [11].

### 2.5 NFACE

The NFACE facility was established (2015 - 2017) to investigate whether under e[CO_2_] wheat cultivar response varied for different levels and types of N input and management and whether this could reverse or slow the known reduction in grain protein and composition under e[CO_2_]. NFACE was located 120 m north of TraitFACE where background soil N was lower, providing the potential for a wheat N response. As with the other AGFACE facilities, there were two levels of [CO_2_] per ring (ambient or 550 µmol/mol). Different levels and types of N were provided and two cultivars known to be more or less efficient in their use of N were sown.

## Ethics Statement

There is no reportable ethics statement in relation to this article publication.

## CRediT Author Statement

**Mahabubur Mollah:** FACE System Engineer and System Manager, Data collection, Data curation, Writing- Original draft preparation; **Glenn Fitzgerald:** Program leadership; Supervision; Experimental design; Funding acquisition; Writing- Reviewing, and Editing.

## Declaration of Competing Interest

We the authors declare that we have no known competing financial interests or personal relationships which have or could be perceived to have influenced the work reported in this article.

## Data Availability

Maybe the solution here is to ask the IsoArch authors to provide more information in the paper about data quality (e.g., additional indeces as R2 mentions) and provide whatever information would be re (Original data) (Mendeley Data).
